# Charting the future: current and future directions in translational research for individuals with Down syndrome

**DOI:** 10.1186/s11689-025-09630-8

**Published:** 2025-07-08

**Authors:** Katherine A. Waugh, Heather M. Wilkins, Keith P. Smith, Lauren T. Ptomey

**Affiliations:** 1 Kansas Intellectual & Developmental Disabilities Research Center, University of Kansas Medical Center, 3901 Rainbow Boulevard, Kansas City, KS 66160 USA; 2 Department of Cell Biology and Physiology, University of Kansas Medical Center, 3901 Rainbow Boulevard, Kansas City, KS 66160 USA; 3 Department of Pediatrics, University of Kansas Medical Center, 3901 Rainbow Boulevard, Kansas City, KS 66160 USA; 4 University of Kansas Alzheimer’s Disease Research Center, 4350 Shawnee Mission Parkway, Fairway, KS 66205 USA; 5 Department of Biochemistry and Molecular Biology, University of Kansas Medical Center, 3901 Rainbow Boulevard, Kansas City, KS 66160 USA; 6 Department of Neurology, University of Kansas Medical Center, 3901 Rainbow Boulevard, Kansas City, KS 66160 USA; 7 Department of Internal Medicine, University of Kansas Medical Center, 3901 Rainbow Boulevard, Kansas City, KS 66160 USA

**Keywords:** Down syndrome, Trisomy 21, Intellectual and Developmental Disability, Precision Medicine, Brain Health, Biomarkers

## Abstract

The most common genetic cause of intellectual and developmental disability is trisomy of human chromosome 21 (trisomy 21) or Down syndrome. Relative to the general population, individuals with Down syndrome heterogeneously experience atypical morphogenesis, a distinct neurocognitive profile, and a unique spectrum of diverse medical conditions that impact every major organ system. How trisomy 21 results in the highly variable manifestations of Down syndrome remains largely unknown and an active area of heavy investigation with therapeutic implications. For example, common inflammatory and metabolic signatures have begun to emerge across various co-occurring conditions in Down syndrome with assorted impacts on diverse yet intertwined organ systems that could directly or indirectly impact brain health. Here, we review current progress, resources, knowledge gaps, and bottlenecks for precision medicine approaches to promote brain health across the lifespan among individuals with Down syndrome within the larger context of research efforts geared towards our other distinct yet intertwined organ systems. Within this framework, we advocate for interdisciplinary pursuit of systems-level biomarkers to facilitate holistic intervention strategies that precisely benefit individuals with trisomy 21 each experiencing Down syndrome in their own unique way. To this end, we quantitatively assess clinical studies that are actively recruiting participants with Down syndrome and provide historical context through summary figures sourced to user-friendly tables that have been curated from federal websites to empower efficient exploration of research opportunities for interdisciplinary collaborations.

**Supplementary Information:**

The online version contains supplementary material available at 10.1186/s11689-025-09630-8.

## Introduction

Down syndrome (DS) is predominately caused by trisomy of human chromosome 21 (trisomy 21, T21), which represents the most common genetic cause of intellectual and developmental disability (IDD) worldwide [1–3]. In the United States, T21 occurs at an estimated rate of 1 in 582 live births, a prevalence that has significantly increased over the years even after adjusting for our concomitant rise in maternal age [2]. Furthermore, the median life expectancy for individuals with DS has dramatically increased, from just 4 years in the 1970 s to approximately 60 years today [4]. However, this remains nearly 20 years below the life expectancy reported for our total United States population and there is a need for enhanced approaches to systematically improve the quality of life among individuals with DS, who are disproportionately impacted by co-occurring medical conditions, such as early-onset of Alzheimer’s disease (AD) [5–9]. Therefore, our research community must continue to refine medical care guidelines for targeted approaches to address the evolving needs of our growing DS population and therefore improve both quantity and quality of life [10–12].

Indeed, hallmarks of DS include numerous co-occurring conditions with highly variable severity and incomplete penetrance [3, 7]. For instance, individuals with DS heterogeneously experience IDD, including atypical morphogenesis, autoinflammation, metabolic dysfunction, and cognitive impairment. Not surprisingly, individuals with DS are also predisposed to a clinical profile that significantly differs from the general population across the lifespan including a lower risk for most solid malignancies alongside an increased risk for leukemias, obesity, diabetes, obstructive sleep apnea (OSA), severe respiratory viral infections (such as RSV in infants and COVID-19 in adults), as well as autoinflammatory and immune disorders [7, 13–15]. The distinct neurological profile experienced by individuals with DS also includes a predisposition to seizures [16] and diagnosis with autism spectrum disorder (ASD) [17] as well as fully-penetrant AD though with highly variable timelines of disease progression to dementia [4]. Therefore, it is not a surprise that a large portion of biomedical research for people with DS has focused on improving brain health across the lifespan.

Brain health is an evolving term defined today by the World Health Organization (WHO) as “the state of brain functioning across cognitive, sensory, social-emotional, behavioral and motor domains, allowing a person to realize their full potential over the life course, irrespective of the presence or absence of disorders (WHO, 2024) [18].” In the general population, many of the co-occurring medical conditions heterogeneously experienced by individuals with DS are interrelated and have been both directly and indirectly linked to brain health. For instance, obesity is a known risk factor for OSA [19], endocrine disorders [20], and cardiometabolic diseases [21], all of which are also associated with an increased risk of AD-related dementia [22–25]. However, the relationships between these factors in individuals with DS remain less understood [26].

While the mechanisms underlying the diverse neurological trajectories in individuals with DS remain largely unknown, research has begun to identify key genetic, environmental, and lifestyle factors that may dynamically influence brain health [7]. A precision medicine approach in healthcare would therefore consider such factors to identify the most effective, personalized treatment options for our highly heterogeneous population of individuals with DS. Recent clinical studies have begun to incorporate measures of genetic, environmental, and lifestyle factors in the context of brain health, offering a timely opportunity to connect cutting-edge preclinical and observational biomarker studies in humans with clinical trials focused on interventions to improve brain health in DS. Here, we review current progress, resources, knowledge gaps, and bottlenecks for precision medicine approaches to promote brain health across the lifespan among individuals with DS.

### Brain health and co-occurring medical conditions across the lifespan in Down syndrome

The National Institutes of Health’s (NIH) INCLUDE (INvestigation of Co-occurring conditions across the Lifespan to Understand Down SyndromE) Project began in June 2018 to support research aimed at improving health and quality life among individuals with DS [27, 28]. This trans-NIH initiative was in direct response to a Congressional directive to dedicate more funds to DS research following the landmark speech by a self-advocate with DS, Mr. Frank Stephens, given to the United States House of Representatives in 2017 where he famously read that “I a man with DS, and my life is worth living [29, 30].”

**Fig. 1:**
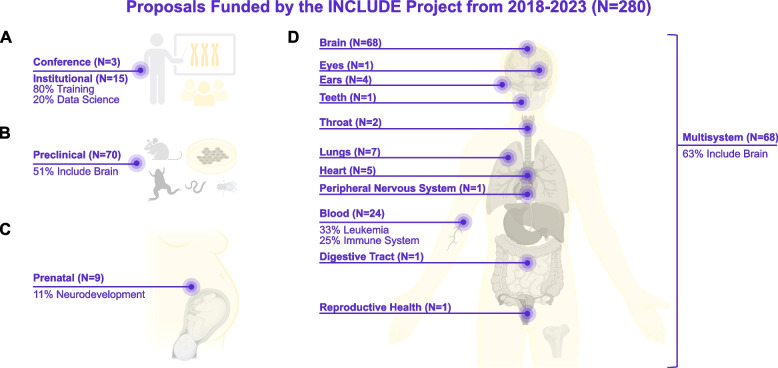
Focus of NIH projects that have received funding through the INCLUDE Project. Schematics summarize studies funded by the NIH INCLUDE Project based on information extracted for all five years listed in September of 2024 from https://www.nih.gov/include-project/funding (Supplemental Table 1). Only one duplicate study from the total of 281 reported was removed (Project Number 3R01DC010290-08S1) to give a total number (N) of 280 funded proposals with 44% listed as supplements to NIH grants that were already funded. Studies were then manually curated by organ (system/s) of study focus using publicly available project information listed across this INCLUDE website and NIH RePORTER tool (https://reporter.nih.gov/). Summary data are provided across diagrams made in BioRender for the following project categories: (**A**) conferences and institutional support, such as funding for the INCLUDE Data Hub or various training grant supplements; (**B**) preclinical studies focused on mice, frogs, worms, flies, and cell lines of any species that do not specify use of primary human participants, samples, or data; (**C**) prenatal or (**D**) non-prenatal human studies that propose to leverage primary human participants, samples, or data

Stated objectives of the INCLUDE Project include the following: 1) advance basic research through funding innovative studies that deepen the understanding of DS and therefore facilitate the development of new therapies; 2) form a large clinical network of diverse individuals with DS interested in research participation by actively enrolling new participants, integrating existing cohorts to increase power, and retaining participation to facilitate longitudinal studies; and 3) promote the participation of individuals with DS in clinical trials by increasing inclusion and establishing assessment standards [28, 31]. Consequently, hundreds of research projects have been funded to explore various health challenges differentially modulated by T21 and underlying mechanisms (Supplementary Table 1). A minority of funds have also been directed to bolstering research infrastructure (e.g., conferences, training grants, and data coordination hubs) (Fig. 1A), and more than half of funds have focused on brain health (Supplementary Table 1).

Relevant preclinical studies have leveraged diverse model systems, that not surprisingly feature mouse models and human induced pluripotent stem cells (hiPSCs) (Table 1), but which also include a minority of innovative studies funded by the NIH INCLUDE Project that leverage frogs, flies, fish, and worm model systems geared at filling key gaps of our basic science knowledge (Fig. 1B) [32]. Notably, supplements to NIH grants that were already funded represent approximately half of all funded projects listed to date on the INCLUDE website (Supplementary Table 1). One such supplement regarding brain health across the lifespan among individuals with DS represents the only prenatal neurodevelopment study funded by the NIH INCLUDE Project to leverage primary human samples (Fig. 1C). This award enables the incorporation of DS samples into recent initiatives aimed at mapping the molecular, cellular, and morphological developmental trajectories of the human fetal [33]. As the majority of high-resolution studies of the aberrant brain development in Down syndrome has been done in preclinical model systems [7], these first-in-kind datasets could provide a valuable reference for both establishing the validity of and ultimately translating numerous preclinical studies on atypical neurodevelopment in DS to facilitate development of early intervention strategies, such as those outlined by Guedj et al*.,* (Supplementary Table 1) [34].

**Table 1:** Preclinical investigation of potential biomarkers for brain health in models of Down syndrome

BIOMARKER	MEASURE	LEVELS, ASSOCIATION (MODEL)	REFERENCES
MRI BRAIN STRUCTURE AND VOLUME	Neurodegeneration	Reduced brain volume (Dp1Tyb); cerebellar microstructural changes and cholinergic circuitry disruption (Ts65Dn)	[35–37]
MRS BRAIN METABOLITES	Brain metabolism	Increased glutamine and reduced taurine (Dp1Tyb); lower glutamine and higher myoinositol (Ts65Dn)	[35, 38]
COGNITIVE TESTS	Memory, learning, and executive function	Impaired learning, reduced visual and spatial integration (Ts65Dn)	[39–42]
CYTOKINES	Inflammation and neuro-inflammation	Increased IL-1b, TNFa, IL-6, G-CSF, and GM-CSF (Ts65Dn)	[43]
OXIDATIVE STRESS MARKERS	Metabolic disorder and inflammation	Elevated (Ts65Dn, Ts1Cje, Dyrk1A overexpression, Ts16)	[44–50]
SYNAPTIC MARKERS (E.G., SYNAPTOPHYSIN)	Neurodegeneration	Loss of cholinergic neurons (Ts65Dn)	[42, 44, 45, 51, 52]
MITOCHONDRIAL MARKERS	Metabolic disease	Reduced brain expression of ETC proteins and reduced ATP (Ts65Dn), reduced function and dynamics (hiPSC), reduced function and ATP (Ts1Cje, Dyrk1A overexpression, Ts16)	[45, 47–50, 53, 54]
ACHE	Neurodegeneration	Reduced in brain (Ts65Dn)	[37]
AD-RELATED BIOMARKERS	AD pathology and neurodegeneration	Elevated Aβ, p-Tau, and NfL (Ts65Dn and Ts1Cje)	[48, 55]
HOMOCYSTEINE	Inflammation and metabolic disease	Elevated (DYRK1A overexpression)	[56]
MONOAMINES	Depression, pain, anxiety	Elevated dopamine and serotonin (Ts1Cje and Ts65Dn)	[57, 58]
BDNF (BRAIN-DERIVED NEUROTROPHIC FACTOR)	Neuronal health	Reduced in frontal cortex (Ts65Dn)	[59]
MOTOR FUNCTION	Coordination and motor control	Abnormal gait (Ts65Dn, Dyrk1a overexpression and Tc1)	[60–62]
NOCICEPTIVE STIMULATION	Pain	Reduced nociception (Ts65Dn)	[63]
THYROID HORMONES	Metabolic disease	Reduced T4 (Dyrk1A overexpression)	[64]

*Abbreviations: Aβ* amyloid beta, *AChE* Acetylcholinesterase, *AD* Alzheimer’s Disease, *ATP* Adenosine triphosphate, *BDNF* Brain-Derived Neurotrophic Factor, *Dp1Tyb* Trisomic for ~ 65% of the genes orthologous to human chromosome 21 mouse model of DS, *Dyrk1A* Overexpression, dual-specificity tyrosine-(Y)-phosphorylation regulated kinase 1a mouse model for DS, ETC Electron transport chain, *GCSF* Granulocyte colony stimulating factor, *GMCSF* Granulocyte–macrophage colony-stimulating factor, *IL1β* interleukin 1β, *IL-6* Interleukin 6, *iPSC* Induced pluripotent stem cells, *MRI* Magnetic resonance imaging, *MRS* Magnetic resonance spectroscopy, *NfL* Neurofilament light, *p-tau* Phosphorylated tau, *T4* Thyroxine, *T65Dn* Trisomic for ~ two-thirds of the genes orthologous to human chromosome 21 mouse model of DS, *Tc1* Trisomic for ~ 90% of the genes orthologous to human chromosome 21 mouse model of DS, *TNFα* Tumor necrosis α, *Ts1Cje* Partial trisomy 16 mouse model for DS, *Ts16* Trisomy 16 mouse model for DS, *BDNF* Brain-Derived Neurotrophic Factor

### Preclinical investigation of brain health biomarkers in models of Down syndrome

Over half of the remaining INCLUDE-supported studies involve the brain and leverage human participants, samples, or data at later developmental stages from infancy into older adulthood (Supplementary Table 1, Fig. 1D). Many are multisystem studies, with 15 of the 68 projects investigating sleep and its impact on brain outcomes. This aligns with precision medicine approaches that consider genetics, environment, and lifestyle to improve brain health—such as enhancing sleep quality to boost cognitive function. Factors like physical health, social connections, and lifelong learning also influence brain development and resilience (WHO, 2024). Lastly, it is worth noting that none of the clinical studies funded by the INCLUDE Project focus on the liver or skeletal system (Fig. 1D). Further exploring the liver-brain axis as well as the relationship between concomitant brain and skull development therefore represent strategies that are currently understudied to improve brain health in DS [65–67].

### Biomarkers of brain health in Down syndrome

Biomarkers are essential to improve brain health in DS through precise treatment strategies that efficiently incorporate an individual’s genetics, environment, and lifestyle (Table 2). According to the FDA-NIH Biomarker Working Group, a biomarker is “a defined characteristic that is measured as an indicator of normal biological processes, pathogenic processes, or responses to an exposure or intervention [68, 69]”. Recent advancements in genetic testing for different DS subtypes, including cases caused by translocation or mosaicism, highlight the importance of genetic testing in precision medicine approaches [70]. While most DS cases result from complete T21 [1], up to 10% of cases have been reported to arise from these alternative genetic events that systematically vary in the severity and penetrance among many of the co-occurring conditions heterogeneously experienced by those with DS [70]. Research on intervention strategies to enhance developmental trajectories in infants and young children with DS remains highly active with a focus on this highly plastic phase of neurodevelopment (Supplemental Table 1). Early interventions are now possible thanks to recent substantial advances in non-invasive prenatal testing (NIPT), which can genetically identify chromosomal anomalies that cause DS using fetal material in maternal circulation [71].

**Table 2:** Brain health biomarkers in Down syndrome

**BIOMARKER**	**MEASURE**	**LEVELS–ASSOCIATION**	**REFERENCES**
PLASMA AND CSF NFL	Neurodegeneration	Elevated—Significantly associated with AD	[72–76]
PLASMA GFAP	Neuroinflammation	Elevated—Associated with cerebrovascular disease	[76–78]
PLASMA BDNF	Neuronal health	Elevated, decreases with age	[79]
S100B PROTEIN	Neuroinflammation	Elevated in amniotic fluid, elevated in serum, not age dependent	[80–83]
PLASMA AND CSF CYTOKINES	Inflammation and neuroinflammation	Elevated	[72, 75]
HOMOCYSTEINE	Inflammation and metabolic disease	Elevated plasma levels with age, lower levels in children—Possible association with some cognitive measures	[84, 85]
MONOAMINES	Depression, pain, anxiety	Elevated CSF 5-HIAA, HVA, and norepinephrine in plasma; higher brain serotonin receptor expression postmortem	[86–89]
MARKERS OF OXIDATIVE STRESS	Metabolic disorder and inflammation	Elevated in saliva and plasma, no change in urine	[90–93]
SYNAPTIC MARKERS (E.G., SYNAPTOPHYSIN)	Neurodegeneration	Reduced in post-mortem brain	[94]
ACHE	Neurodegeneration	Reduced in postmortem brain	[95, 96]
THYROID HORMONES	Metabolic disease	Elevated in serum/plasma	[97–99]
BRAIN VOLUME AND WHITE MATTER INTEGRITY	Neurodegeneration	Reduced brain volume and cortical thickness; increased markers of white matter integrity loss; increased parahippocampal gyrus volume—Possible association with some cognitive measures	[100–102]
MITOCHONDRIAL MARKERS	Metabolic disease	Elevated mitochondrial mass and oxidative stress in fetal samples, reduced expression of ETC proteins and increased mtDNA mutations/deletions in postmortem brain	[103–105]
COGNITIVE TESTS	Memory, learning, and executive function	Validated measures include CS-DS, CAMDEX-DS	[72, 106–111]
QUANTIATION OF PAIN	Pain	Those with DS may have difficulty reporting pain. Validated measures include NCCPC-R, PPP, r-FLACC, INRS, COMFORT-B, and quantitative sensory testing	[112–115]
FDG-PET	Brain glucose metabolism	Reduced in the posterior cingulate and parietotemporal cortex of the brain	[116–119]
MOTOR FUNCTION	Coordination and motor control	Validated measures include BMS, GMFM, MOT, Movement ABC, TWT	[120–123]

Biomarker definition and levels are listed relative to general population with associated outcomes among individuals with Down syndrome

*Abbreviations: 5-HIAA* 5-Hydroxyindoleacetic acid and the main breakdown product of serotonin, *HVA* Homov vanillic acid (HVA) a metabolite of dopamine, *AChE* Acetylcholinesterase, *BDNF* Brain-Derived Neurotrophic Factor, *BMS* Basic motor skills, *CAMDEX-DS* Cambridge Examination for Mental Disorders of Older People with Down's syndrome and Others with Intellectual Disabilities, *COMFORT-B* COMFORT-Behavior Scale, *CS-DS* Cognitive Scale for Down Syndrome, *CSF* Cerebral spinal fluid, ETC Electron transport chain, *FDG-PET* Fluorodeoxyglucose positron emission tomography, *GFAP* Glial fibrillary acidic protein, *GMFM* Gross Motor Function Measure, *INRS* Individualized Numeric Rating Scale, *MOT* Motor screening task, *Movement ABC* Movement Assessment Battery for Children, *mtDNA* Mitochondrial DNA, *NCCPC-R* Non-communicating Children’s Pain Checklist-Revised, *NFL* Neurofilament Light, *PPP* Pediatric Pain Profile, *r-FLACC* Revised Face, Legs, Activity, Cry and Consolability Scale, *TWT* Trail walking task

### Brain health biomarkers in Down syndrome

In addition to diagnostic biomarkers, subtypes also include predictive, prognostic, pharmacodynamic, susceptibility/risk, monitoring, and safety biomarkers, that each require precise definitions and protocols [69]. All biomarkers must be validated to support regulatory approval. Safety biomarkers, for instance, are vital to monitor for drug toxicity in liver, kidney, or cardiovascular function during clinical trials. However, although these biomarkers have been validated in the general population [69], systematic study for many of these markers is lacking among people with DS [124]. For example, while biomarkers of liver function are often included in blood analyses (e.g., aspartate transaminase and alanine transaminase), their relevance to health and disease in DS has become unclear [124–129]. Lastly, another common issue in biomarker evaluation is assuming that correlation with clinical outcomes qualifies a biomarker as a surrogate measurement [69]. For a biomarker to be a valid surrogate, changes in the biomarker must directly explain changes in clinical outcomes, and therefore requires rigorous study as reflected in our manually curated tables of established biomarkers (Tables 2 and 3) [68].

**Table 3:** Alzheimer’s disease biomarkers in Down syndrome

**BIOMARKER**	**MEASURE**	**LEVELS–ASSOCIATION**	**REFERENCES**
PLASMA AMYLOID (AΒ_42_/AΒ_40_)	Blood (plasma) levels of Ab ratios. Ratios are reduced in AD	Reduced—Possible association with some cognitive measures	[72–74, 76, 78, 130]
AMYLOID PET	Positron Emission Tomography of brain amyloid plaques. Levels elevated in AD	Elevated—Similar accumulation rates to sporadic AD	[131–135]
CSF AMYLOID (AΒ_42_/AΒ_40_)	CSF levels of Ab ratios. Ratios reduced in AD	Reduced	[75, 136]
PLASMA TAU (T-TAU, PTAU217)	Blood (plasma) levels of t-Tau and p-Tau. Levels elevated in AD	Elevated—Possible association with some cognitive measures	[73, 76, 78, 132, 134]
TAU PET	Positron Emission Tomography of brain tau tangles. Levels elevated in AD	Elevated following positive amyloid PET	[132, 135, 137]
CSF TAU	CSF levels of Tau. Levels elevated in AD	Elevated	[75, 138]

Biomarker definition and levels are listed relative to general population with associated outcomes among individuals with Down syndrome

*Abbreviations: Aβ* Amyloid beta, *CSF* Cerebral spinal fluid, *PET* Positron emission tomography, *p-Tau* Phosphorylated Tau, and *T-tau* total tau

**Fig. 2:**
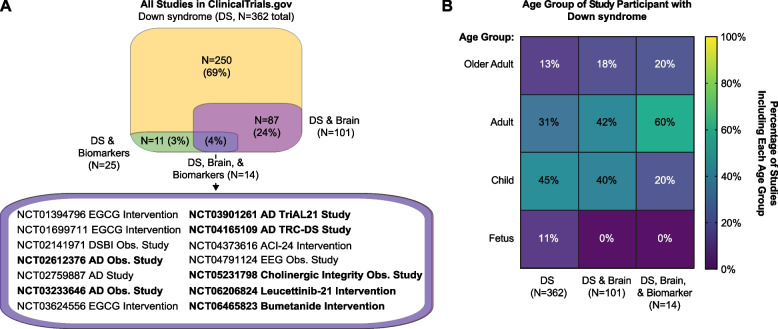
Few clinical studies of Down syndrome feature both the brain and biomarkers. **A** Top: Venn Diagram of subset overlap within a total of 362 studies in https://clinicaltrials.gov/ that contain the Condition/Disease “DS” or Other Term “T21” in August 2024 (Supplemental Table 2, Tab 1). Of those 362 studies, 25 have the term “biomarker” listed anywhere in a Data Field and 101 have an outcome measure directly related to the brain. Bottom: ClinicalTrial.gov Identifiers for the 4% of studies that relate to DS containing both the term biomarker and a brain outcome measure with active studies in bold. **B** Age group of DS study participants among study subsets outlined in “A” and colored by percentage of study subset that is included in each age group either specified by ClinicalTrials.gov (i.e., Child, Adult, and/or Older Adult) or labeled as “Fetus” if one of the following terms is in a Data Field then manually curated to confirm: prenatal, pregnant, pregnancy, fetus, fetal, and maternal. The heatmap was made using GraphPad Prism v10.3.1 on data curated from ClinicalTrials.gov in August 2024 (Supplemental Table 2, Tab 1)

To explore the cutting-edge efforts underway to augment brain health in people with DS, we next leveraged publicly available data in ClinicalTrials.gov. Of the 362 DS studies registered on ClinicalTrials.gov, 25 focus on biomarkers, and 101 have brain-related outcomes (Fig. 2A). Fourteen active studies overlap between these two areas with half observational and half testing interventions (Fig. 2A). Among these, three interventional studies to improve cognition and reduce neurodegeneration aim to target chromosome 21-encoded DYRK1A (Dual specificity tyrosine-phosphorylation-regulated kinase 1 A) through the nutraceutical epigallocatechin-3-gallate in green tea extract, and one trial focuses on an anti-AD vaccine, ACI-24 (Supplementary Table 2, Tab 1). Notably, 9 of the 14 clinical studies focus on neurodegeneration in DS. One example is the DS Biomarker Initiative (DSBI), a pilot study that followed 12 adults with and without dementia using MRI and PET imaging to correlate brain changes with cognitive decline (NCT02141971) [139]. This study laid the groundwork for larger cohort studies like the Trial Ready Cohort – DS (TRC-DS, NCT04165109) and the Alzheimer’s Biomarker Consortium – DS (ABC-DS) [140], from which a recent review has been published summarizing progress in biomarker identification specifically from the blood [141]. Indeed, biomarkers of AD in DS has become a fast-moving field. Notably, such progress has resulted from international efforts across the globe with researchers all working towards this same goal. These clinical studies are not always listed on ClinicalTrials.gov which is run through United States [142].

It should also be noted that only studies that explicitly list “biomarkers” in study descriptions are considered in this review as biomarker studies registered with ClinicalTrials.gov (Supplementary Table 2, Tab 1). This conservative approach highlights registered studies focused on novel biomarker discovery or validation but likely exclude brain health studies that incorporate established biomarkers that are already commonly applied to other contexts, such as AD, OSA, or even safety. In addition, many observational studies related to biomarker development in DS are not registered on ClinicalTrials.gov (e.g., ABC-DS) as it is optional for non-interventional studies.

Nevertheless, clear gaps restrict progress towards incorporating precision medicine approaches for brain health across the lifespan of individuals with DS (Supplementary Table 2, Tab 1). First, most clinical studies focus on adults, leaving prenatal neurodevelopment underexplored (Fig. 2B). Additionally, few clinical studies have examined environment and modifiable lifestyle factors which may play an important role in brain health in persons with DS (Supplementary Table 2, Tab 1). For example, a 2024 Lancet report [25] indicates that nearly half of all dementia cases worldwide could be prevented or delayed by addressing 14 modifiable risk factors including education level, LDL-cholesterol, physical inactivity, diabetes, obesity, and social isolation. Thus, biomarker discovery and validation related to lifestyle and environmental factors is critical.

### Clinical research focused on Down syndrome

**Fig. 3:**
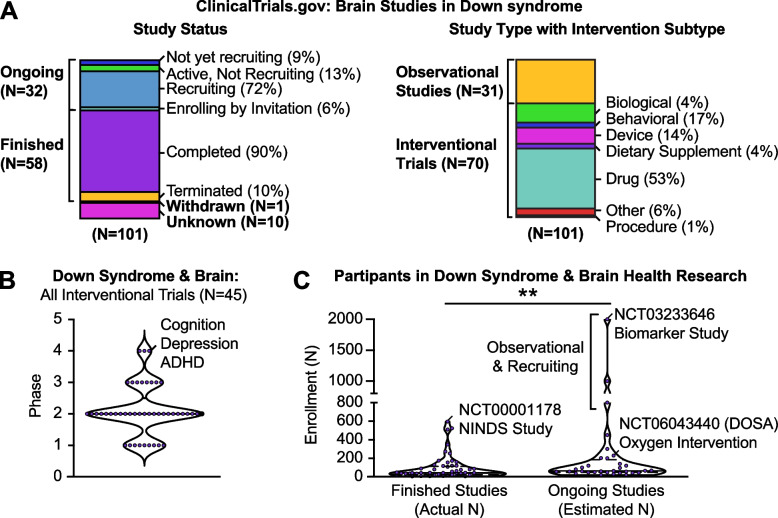
Demographics of research studies for people with Down syndrome that contain outcomes directly relating to the brain (*N* = 101 studies, Supplemental Table 2, Tab 2). **A** Left: Status of study on ClinicalTrials.gov. 32 studies are ongoing with a portion of those (%) listed as either active, not recruiting; recruiting; enrolling by invitation; or not yet recruiting. 58 studies are finished with a portion of those (%) listed as either completed or terminated. A total of 10 studies have unknown status and only 1 study has been withdrawn. Right: Type of study on ClinicalTrials.gov. 31 studies are listed as observational and 70 trials are listed as interventional following manual curation with a portion of those (%) interventions categorized as biological, behavioral, device, dietary supplementation, drug, other, or procedure. **B** Each interventional trial in “A” with phase listed on ClinicalTrials.gov is represented within a violin plot (purple dot, *N* = 45). When more than one phase is assigned to a study, all phases were included as separate data points. Key terms are listed for all three of the Phase 4 clinical trials. **C** Violin plots represent the number (N) of participants enrolled in each of the finished or ongoing studies listed in “A” with **p < 0.01 by Mann–Whitney. **A-C** Demographics are listed as they appeared on ClinicalTrials.gov in August 2024. Nevertheless, the study sponsor or investigator is responsible for the safety, science, and accuracy of any information submitted to ClinicalTrials.gov (https://clinicaltrials.gov/about-site/disclaimer). All graphs were made using GraphPad Prism v10.3.1on data manually curated from ClinicalTrials.gov in August 2024 (Supplemental Table 2, Tab 2)

The current breadth and depth of clinical studies focused on DS is unprecedented; exactly half of the 14 total studies that feature biomarkers and brain health have an active status with 1 to be completed December 2024 and 3 more in 2025 (Supplementary Table 2, Tab 2). In addition, 36% of the 101 clinical studies on brain health in DS are ongoing with roughly ¾ including an intervention (Fig. 3A). Most of these clinical trials that have been assigned a phase are currently active or have stalled (completed/terminated) in Phase 2 while defining the effectiveness or lack of an experimental intervention to facilitate brain health in DS (Fig. 3B). A total of 3 clinical trials qualify as Phase 4 under therapeutic development to define side effects caused over time by a new treatment on the market after approval (Fig. 3B). These include pharmacologic treatments for cognitive impairment, depression, and attention deficit/hyperactivity disorder (ADHD) in individuals with DS (Supplementary Table 2, Tab 2). Specifically, the first DS trial to enter Phase 4 in 2008 involved the use of an N-methyl-D-aspartate (NMDA) receptor uncompetitive antagonist, memantine hydrochloride (i.e., NAMENDA) to improve cognition in a cohort of 42 adults with DS (Supplementary Table 2, Tab 2). As memantine was already approved for treating moderate-to-severe AD (NCT01112683), the reported results were disappointing upon trial completion in 2011 with a lack of efficacy now confirmed across multiple clinical trials [143, 144]. The next DS trial to enter Phase 4 involved preliminary assessment of whether fluoxetine (i.e., PROZAC) is effective, safe, and tolerable in 6 adults total with DS to treat depression [145]. This trial was recently completed in February 2024 but has yet to report results (NCT05458479). Lastly, an ongoing pilot study of the Quillivant stimulant in 30 children with DS and ADHD (NCT04219280) will be completed in 2025. It will be interesting to see how biomarkers are incorporated into results reported from these clinical trials.

Most of the clinical research on brain health in DS includes qualifiers in their project descriptions that reflect low power from reliance on smaller proof-of-principal cohorts, such as “exploratory,” “preliminary,” or “pilot” (Supplementary Table 2, Tab 2). On ClinicalTrials.gov, the largest clinical study to date of brain health in DS was registered under the NIH’s National Institute of Neurological Disorders and Stroke (NINDS) to assess frontal lobe dementia as well as Parkinsonian disorders, which recruited 597 people total over 32 years until study termination in in 2012 (NCT00001178) (Fig. 3C). However, the relevance of this study to brain health in DS remains unconfirmed as no results have been posted on ClinicalTrials.gov ID or published that specifically cite this unique identifier and DS was not the sole focus (Supplementary Table 2, Tab 2).

Nevertheless, developing large cohorts of participants with DS willing to participate in longitudinal research is essential to power deep phenotyping and natural history studies. The cohort development component of the NIH INCLUDE Project is designed to build a large, diverse cohort of individuals with DS across their lifespan, enabling the long-term study of co-occurring conditions, such as AD, autoimmune disorders, and cardiovascular issues, which affect individuals with DS at higher rates. Not surprisingly, ongoing clinical studies of brain health in DS are anticipated to be significantly larger than previous studies that have been completed/terminated (Fig. 3C).

The largest of these ongoing studies registered on ClinicalTrials.gov aims to recruit 2000 people and thereby develop and evaluate innovative biomarkers of brain health through non-invasive measures of retinal and choroidal microvasculature (NCT03233646). The interventional study anticipated to recruit the most participants represents a Phase 2 trial to assess supplemental oxygen during sleep and will apply an outcome to assess working memory among 230 children with DS and OSA (DOSA, NCT06043440).

Notably lacking in these ClinicalTrials.gov data is the ABC-DS which represents the largest cohort specific to brain health in DS (*N* = 450 and growing) [140]. The ABC-DS was funded in 2020, to establish clinical, cognitive, genetic, ‘omics, and imaging biomarkers of AD in DS. The ABC-DS merges two cohort studies conducted simultaneously since 2015: Neurodegeneration in Aging DS (NiAD, U01AG051406) and AD in DS (ADDS, U01AG051412). This combined cohort represents the largest cohort of AD in DS, with 10 sites distributed across the United States (9 sites) and the United Kingdom (1 site). With over 83 associated publications to date, ABC-DS has been pivotal in the development and validation of biomarkers used across diverse clinical studies of brain health in DS [146], including cognitive outcomes like the modified Cued Recall [147]. Additionally, several supplements and ancillary R01 projects are linked to ABC-DS, adding measures related to environmental stress and lifestyle (e.g., sleep and physical activity, R01AG070028), maternal genetic factors related to AD (U19AG068054-03S1), and walking gait (U19AG068054-04S2). These projects contribute to the development of precision medicine approaches. For example, recent findings indicate that greater physical activity is associated with better cognitive performance across multiple domains in DS [148].

While ABC-DS has become an instrumental resource to further biomarker development for AD in DS as well as to expand our understanding of AD etiology and pathology timing in DS, similar efforts are needed to recruit large, diverse, and deeply-phenotyped cohorts during prenatal care, childhood, adolescence, and early adulthood. For example, susceptibility to racial inequalities among individuals with DS shows pronounced differences that significantly varies across the lifespan and is more pronounced than what is seen in the general population, particularly in infancy and middle-age [149]. These efforts to increase cohort number and diversity would help shed light on the mechanisms driving the high risk of comorbid conditions relevant to brain health that arise both in utero and across the lifespan. To meet these needs the INCLUDE project has launched the DS Cohort Development Program in late 2024, with the goals of improving our understanding of the natural history of DS and the relationship of demographic factors (e.g., race, ethnicity, gender, geographic location) to outcomes; exploring factors related to the development of critical and co-occurring health conditions for individuals with DS; and expanding our understanding of the safety, dosing and efficacy of therapeutics across the lifespan.

## Surge in clinical research focused on Down syndrome

**Fig. 4:**
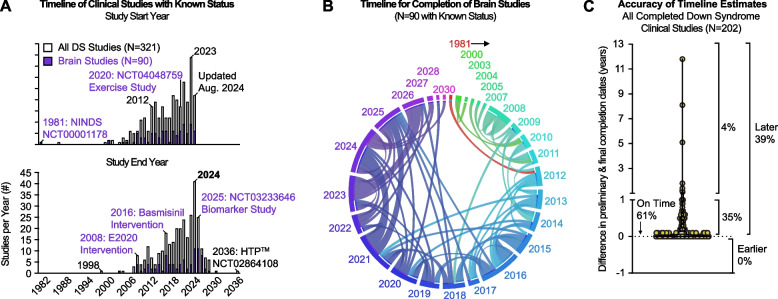
Timeline of clinical studies with a Down syndrome focus. **A** After excluding studies with a status of unknown or withdrawn, clear bars in graph represent the number, 321, of the 362 DS studies in ClinicalTrials.gov with a known study status by study start (top) or end (bottom) year. Of those 321 studies, 90 studies are highlighted in purple that have an outcome measure directly related to the brain. **B **Circos plot [156]. represents DS studies in ClinicalTrials.gov that contain a brain outcome measure with a known study status (*N* = 90). Each study year in the outer circle is represented in numerical order by distinct sectors. The outer circle and connecting track line color are matched to represent study start year; the inner concentric circle color represents study end year which is connected to the study start year by the track line. Size of tracks are scaled to number of studies with more studies being represented by a larger connecting track line. **A-B** Study start and end years are based on actual dates for completed studies and estimated dates for ongoing studies (Supplemental Table 2, Tab 2). **C** Difference between preliminary and final completion dates listed for the 202 studies with completed status of the 362 total in ClinicalTrials.gov that contained the Condition/Disease “DS” or Other Term “T21” (Supplemental Table, Tab 1). **A**, **C** Graphs were made with GraphPad Prism v10.3.1 from data in Supplemental Table 2 curated from ClinicalTrials.gov in August 2024

Before Frank Stephen’s catalyzing speech in 2018, DS has historically been the least funded genetic condition on a per person basis [150]. In the past decade the number of studies on ClinicalTrials.gov that include individuals with DS has more the doubled (Fig. 4A), with the quantity and content of studies exhibiting striking fluctuations in concordance with current events (e.g., COVID-19 pandemic), activities of non-profit organizations that are powered by self-advocates with DS and their families (e.g., Global Down Syndrome Foundation), and NIH priorities. For example, a distinct rise in clinical research can be traced to the early 2000s (Fig. 4A), which coincides with the NIH Director’s request for the Eunice Kennedy Shriver National Institute for Childhood Development (NICHD) to form a DS Working Group in 2006 [151]. Another visual spike in clinical studies occurs in 2012 (Fig. 4A), just after the NICHD established the DS Consortium to unite governmental and private organizations, healthcare providers, and self-advocates [152]. That same year, DS-Connect® was launched, an online platform to link individuals with DS to research studies via surveys that collect health data [152]. DS-Connect® is currently being revamped under the relatively new INCLUDE Data Coordinating Center (DCC), with operations temporarily paused in the interim [153]. Once relaunched, its integration with ClinicalTrials.gov and the INCLUDE DCC would facilitate more comprehensive insights into ongoing studies for researchers and participants. Notably, the DS Consortium currently also lists a subset of over 20 clinical studies that are active, with additional details available through links to ClinicalTrials.gov (Supplemental Table 2, Tab 1) [154], many of which are expected to conclude by the end of 2024 (Fig. 4B). As 96% of clinical studies that include people with DS show study completion within one year of the preliminary completion dates (Fig. 4C), it is anticipated that the results from an unprecedented number of clinical trials focused on brain health in DS will be released within the year. Lastly, other organizations, such as LuMind IDSC which focuses on advancing treatments and care for DS, also lists a subset of about 20 active clinical studies on their website that are available for enrollment through links to ClinicalTrials.gov (Supplemental Table 2, Tab 1) [155].

Although only a portion of these ongoing or recently completed research studies specifically focus on biomarker development, many include outcomes related to brain health (Figs. 2A and 4B). For example, a recently completed trial (NCT04048759) examined the impact of a remote exercise program on daily physical activity and cognitive function in 81 young adults with DS with a study design that was exceptionally amenable to trial participation during the years that spanned COVID-19 quarantines (Fig. 4A) [157–159]. Notably, between 2018–2024 the INCLUDE Project funded 3 clinical trials directly related to brain health and are currently on-going. These include: an anti-amyloid treatment trial (NCT04165109), a trial examining the impact of recombinant human granulocyte–macrophage colony-stimulating factor (GM-CSF) on cognitive outcomes (NCT05482334), and a lifestyle intervention examining the impact of weight loss on biomarkers associated with AD (NCT05985486) (Supplemental Table 2, Tab 2).

### Coordinating data release from clinical studies

**Fig. 5:**
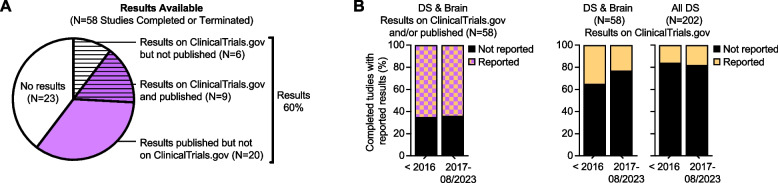
Results from Down syndrome studies in ClinicalTrials.gov. **A** Pie chart and (**B**) bar graph of the 58 studies with a status of completed among the 101 studies on ClinicalTrials.gov that contained the Condition/Disease “DS” or Other Term “T21”, and an outcome measure directly related to the brain. **A** Subsets are striped by result availability on ClinicalTrials.gov and shaded based on result publication following PubMed search for Clinical Trial Identifier. **B** Bar graphs of completed DS studies on brain health in ‘A’ broken-out by pre- or post-NIH mandate to post results on ClinicalTrials.gov within one year of trial completion. **A-B** All graphs were made using GraphPad Prism v10.3.1 on data curated from ClinicalTrials.gov in August 2024 (Supplemental Table 2, Tab 2)

Timely release of results from clinical research is crucial for advancing biomedical research, yet historically there has been a dearth of reported data (Supplemental Table 1, Tab 2). Only 60% of registered clinical studies with brain outcomes posted results on ClinicalTrials.gov or published findings upon completion or termination (Fig. 5A). In 2016, the NIH implemented a policy requiring all NIH-funded clinical trials to register and report results on ClinicalTrials.gov within one year of completing primary data collection (The Clinical Trial Registration and Results Information Submission Regulation at 42 CFR Part 11) [160]. However, this policy change does not correspond to increased frequency of results released from DS studies registered on ClinicalTrials.gov (Fig. 5B).

To further improve data sharing in DS research in more recent years, the NIH-funded an INCLUDE Data Coordinating Center (DCC) [161]. The INCLUDE DCC boasts a secure cloud-based platform for sharing study data across clinical studies with DS cohorts, with 13 harmonized studies that cumulative include 9,421 participants, 44,200 biospecimens, and 113 TB of data so far. Key participating studies include the ABC-DS, the DS-Connect® (NCT01950624), with survey data from 3,634 participants, and the Crnic Institute Human Trisome Project Biobank (HTP™, NCT02864108), which has deposited the largest number of samples and data to date. Importantly, the INCLUDE DCC also hosts workshops and webinars to facilitate dataset access for the wider community of diverse DS researchers.

Not surprisingly, the past four years have witnessed a surge in data integration across multiple high-throughput technologies applied to the same large cohort of individuals with DS [103, 162–164]. These studies often leverage resources registered with the INCLUDE DCC, and cover diverse topics that range from single-cell maps of human fetal blood in DS to monitoring systemic responses to an immunomodulatory intervention among adults with DS [103, 164]. Such investigations often utilize machine learning methods to identify essential features across datasets and offer a unified strategy for discovery across various biological levels to provide a holistic perspective on human health and not surprisingly include facilitating system-level biomarker identification [140, 162–165]. Recent successes in this regard, for which AD-DS studies are at the forefront (Table 3) [140, 146], reflect close interdisciplinary coordination across multiple sites and diverse teams that include but are not limited to clinical coordinators, wet-lab researchers, data scientists and bioinformaticians, and not to mention an extensive commitment by the research participants themselves.

### Alzheimer’s disease biomarkers in Down syndrome

Despite such progress, key challenges still exist for accessing and integrating results across clinical studies including proprietary restrictions and study design limitations. For instance, a recent multi-omic study leveraged whole blood of 304 individuals with DS registered with the INCLUDE DCC through the HTP™ to delineate sub-cohorts enriched for signatures of autoinflammation, as well as aberrant metabolic and immune function [163]. Key elements of aggregate signatures were shockingly stable over time across longitudinal sample collection. Even though these pathways are of high relevance to brain health in DS (Tables 1, 2 and 3) [166], outcomes of cognition and behavior were outside the scope of this first-in-kind study that is also not yet powered alone for biomarker discovery and validation across co-occurring conditions that directly relate to brain health (e.g., ASD and AD). In addition, proprietary restrictions impede interpretation of these data within the larger context of complementary studies of brain health in DS that also source to cohorts registered with the INCLUDE DCC, such as seminal publications that detail relevant metabolic alterations in DS-AD [167]. Promising advances in data sharing include increased study participation in the INCLUDE DCC to facilitate dataset harmonization and shared access among complimentary clinical studies.

## Conclusions

Precision medicine considers an individual’s genetics, environment, and lifestyle to identify the most effective, personalized treatment options. Over the past twenty years, there has been a marked increase in research aimed at promoting brain health in individuals with DS, including prenatal genetic screening to facilitate early intervention strategies, progress towards the development and validation of biomarkers related to diverse aspects of brain health, inclusion and coordinated recruitment of participants with DS to power clinical studies, and the initiation of crucial clinical trials to test promising interventions. While this surge in DS research has brought us tantalizingly close to a precision medicine approach for improving brain health among individuals with DS, we are not there yet.

Quantitative analysis of diverse clinical studies posted on the NIH website for the INCLUDE Project and ClinicalTrials.gov highlight key gaps in current efforts. In the future, clinical research must prioritize incorporating novel biomarkers into studies focused on brain health, validating biomarkers related to environment and lifestyle for integration into these trials, developing biomarkers cut-off points for clinical diagnosis, and establishing large longitudinal cohorts of youth with DS. Additionally, it is essential for researchers to prioritize the publication of results and be open to public data sharing that leverages the collective perspective of diverse DS researchers.

Within our current infrastructure to facilitate DS research, we are optimistic this coming decade of research will address these gaps to significantly advance the field. Nevertheless, it is worth noting that many of the funding mechanisms have not yet been renewed, or cancelled, through which the NIH INCLUDE Project has invested in research to benefit individuals with DS. Delays to NIH website updates have also restricted the scope of this review to studies that have been funded until 2023 as data for 2024 and 2025 have yet to be posted in May 2025. As this quantitative review clearly highlights co-occurring surges in biomedical breakthroughs with targeted investment by the NIH, continuation of the NIH INCLUDE Project is essential for individuals with DS to lead longer, healthier lives.

## Supplementary Information

Supplementary Material 1: Table 1: Projects funded through the NIH INCLUDE Project. List of studies funded by the NIH INCLUDE Project based on information extracted for all five years listed in September 2024 from https://www.nih.gov/include-project/funding. Only one duplicate study from the total of 281 reported was removed (Project Number 3R01DC010290-08S1) to give a total number (N) of 280 funded proposals. Red font denotes edits, additions, and typo corrections to the raw output. Studies were manually curated by organ (system/s) of study focus using publicly available project information listed across this INCLUDE website and NIH RePORTER tool (https://reporter.nih.gov/) into the following project categories: conferences and institutional support, such as funding for the INCLUDE Data Hub or various training grant supplements; preclinical studies focused on mice, frogs, worms, flies, and cell lines of any species that do not specify use of primary human participants, samples, or data; prenatal or non-prenatal studies that propose to leverage primary human participants, samples, or data.

Supplementary Material 2: Table 2: Clinical studies of Down syndrome. A total of 362 studies were exported from ClinicalTrials.gov that match search criteria for the Condition/Disease “DS” or Other Term “T21” in August 2024. Of those 362 studies, 25 have the term “biomarker” listed anywhere in a Data Field and 101 have an outcome measure directly related to the brain. Age group of DS study participants among study subsets were labeled as “Prenatal” if one of the following terms occurred in a Data Field then manually curated to confirm relevance: “prenatal, pregnant, pregnancy, fetus, fetal, and maternal.” Tab 1 contains all studies and Tab 2 contains a subset of those that have an outcome measure directly related to the brain. Tab 2 was manually curated to confirm correct output of “Intervention” Study Type. Please note that the study sponsor or investigator is responsible for the safety, science, and accuracy of any information submitted to ClinicalTrials.gov (https://clinicaltrials.gov/about-site/disclaimer).

## Data Availability

No datasets were generated or analysed during the current study.
